# Balanced Ambipolar Charge Transport in Phenacene/Perylene Heterojunction-Based Organic Field-Effect Transistors

**DOI:** 10.1021/acsami.0c20140

**Published:** 2021-02-15

**Authors:** Tomoya Taguchi, Fabio Chiarella, Mario Barra, Federico Chianese, Yoshihiro Kubozono, Antonio Cassinese

**Affiliations:** † Research Institute for Interdisciplinary Science, Okayama University, Okayama 700-8530, Japan; ‡ CNR-SPIN, c/o Dip. di Fisica “Ettore Pancini”, P.le Tecchio, 80, I-80125 Napoli, Italy; § Dip. di Fisica “Ettore Pancini”, Università “Federico II”, P.le Tecchio, 80, I-80125 Napoli, Italy

**Keywords:** organic semiconductors, field-effect transistors, heterojunction, charge transfer, ambipolar response, film growth mode, vacuum deposition, scanning Kelvin probe microscopy

## Abstract

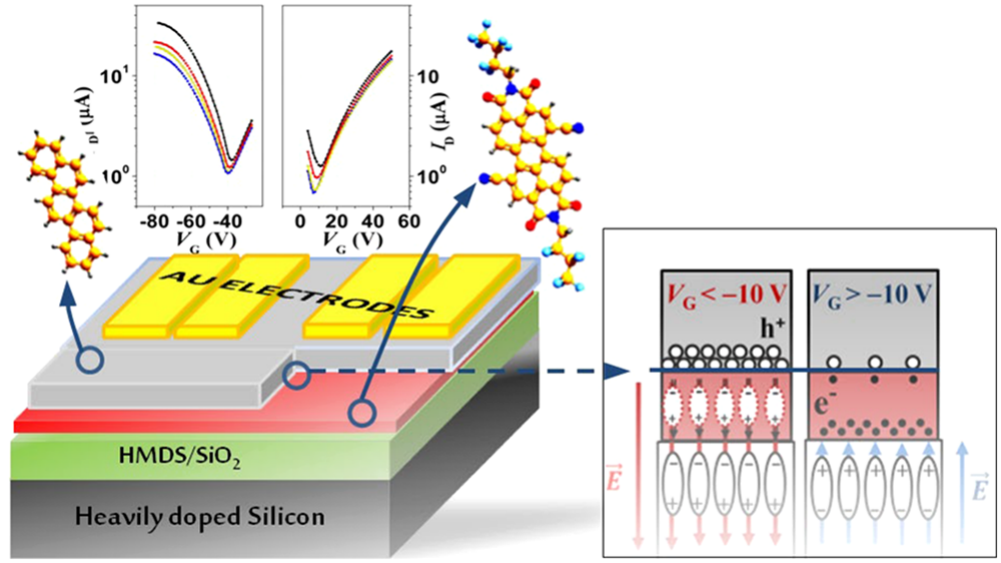

Electronic devices relying on the combination of different conjugated organic materials are considerably appealing for their potential use in many applications such as photovoltaics, light emission, and digital/analog circuitry. In this study, the electrical response of field-effect transistors achieved through the evaporation of picene and PDIF-CN_2_ molecules, two well-known organic semiconductors with remarkable charge transport properties, was investigated. With the main goal to get a balanced ambipolar response, various device configurations bearing double-layer, triple-layer, and codeposited active channels were analyzed. The most suitable choices for the layer deposition processes, the related characteristic parameters, and the electrode position were identified to this purpose. In this way, ambipolar organic field-effect transistors exhibiting balanced mobility values exceeding 0.1 cm^2^ V^–1^ s^–1^ for both electrons and holes were obtained. These experimental results highlight also how the combination between picene and PDIF-CN_2_ layers allows tuning the threshold voltages of the p-type response. Scanning Kelvin probe microscopy (SKPM) images acquired on picene/PDIF-CN_2_ heterojunctions suggest the presence of an interface dipole between the two organic layers. This feature is related to the partial accumulation of space charge at the interface being enhanced when the electrons are depleted in the underlayer.

## Introduction

In the field of organic electronics, despite rapid progress, fundamental challenges must be still overcome to make organic electronic devices commercially available.^1^ One issue is the fabrication of air stable and reproducible ambipolar organic field-effect transistors (OFETs) with balanced hole and electron transport properties, desirable for the realization of complementary inverters.

To this aim, two possible challenging approaches are available:^2,3^ designing a single ambipolar material^4−8^ or composing heterojunctions (i.e., blending^9−12^ or heterostructuring two different organic compounds^13−18^). Materials for ambipolar transistors based on small molecules (e.g., diketopyrrolopyrrole (DPP),^19^ naphthalene diimide (NDI),^20^ isoindigos,^21^ and (*E*)-[3,3′-bipyrrolylidene]-2,2′(1*H*,1′*H*)-dione (BPD)^22^ derivatives) display hole and electron mobility values that typically do not exceed 10^–1^ cm^2^ V^–1^ s^–1^ in ambient conditions with in many cases a poor balance between the two carrier types. Charge-transfer complexes can also be used for ambipolar transport, and recently, donor (silylethynylated pentacene)–acceptor (silylethynylated tetraazapentacene) organic blending semiconductors with a novel form of solid solution were proposed with mobilities of 0.02 and 0.05 cm^2^ V^–1^ s^–1^ for hole and electrons, respectively.^23^ Better results, mobility exceeding 1 cm^2^ V^–1^ s^–1^, are obtained with conjugated polymers and copolymers.^24,25^ In the field of bilayer-based transistors, Chang et al.^26^ demonstrated well-balanced carrier mobility values of 1 cm^2^ V^–1^ s^–1^ in an oxygen-free atmosphere by utilizing ω-diperfluorohexylquaterthiophene (DFH-4T) and dinaphtho[2,3-*b*:2′,3′-f]thieno[3,2-*b*]thiophene (DNTT) as n- and p-type components, respectively. In the bilayer structures, various design factors such as the selection of p- and n-type materials, deposition order, relative film thickness, surface morphology and microstructure of the active layers, source/drain contacts, and p/n interface affect the ambipolar performance. In the past years, different manufacturing approaches have been used: for example, orthogonal solution processes, single-crystal heterostructuring, vacuum vapor deposition, and so on. In this context, the sequential *in situ* deposition process represents an effective strategy to obtain high quality films and interfaces. Structures of sequentially deposited semiconducting layers allow a fine control of the film microstructure and the physical separation of conductive channels for holes and electrons in different regions. On the other hand, the search for the best combination of materials is crucial being necessary to take into account several different parameters such as alignment of energy levels, ambient stability, morphology, optimization of injection and transport properties, and the control of molecular orientation.

In the past decade, phenacenes were demonstrated to be a very interesting molecular family for the fabrication of p-type field-effect transistors displaying remarkable charge transport properties in ambient conditions.^27^ Phenacenes are characterized by a one-dimensional conjugated configuration, where benzene rings are fused in a zigzag (W-shaped) pattern. This specific molecular arrangement provides these compounds with larger band gaps and deeper frontier molecular orbital energy levels in comparison with the “acenes” family (i.e., tetracene and pentacene) which, on the contrary, consist of linearly fused benzene rings. Picene, with five benzene rings, a band gap (*E*_g_) of 3.3. eV, and a HOMO level located at −5.5 eV, was the first member of the phenacenes family to be investigated in relation to its field-effect response and in combination with various dielectric surfaces.^28,29^ Picene thin-film transistors can be fabricated by evaporation techniques, both based on the Knudsen cell and supersonic molecular beam,^30^ and show typically p-channel response with charge carrier mobility up to 1 cm^2^ V^–1^ s^–1^. The charge transport properties of these devices were also demonstrated to be enhanced when they are stored in an oxygen-rich atmosphere. Such behavior was explained in terms of a trap-reduction model, describing the trap density reduction upon the O_2_ reaction with the trapping centers. Based on this oxygen-sensing capability, picene transistors were considered in view of the possible application as O_2_ gas sensors.^31^

Similar to other phenacenes, picene vacuum-deposited thin films were shown to exhibit a favorable molecular arrangement on more hydrophobic (i.e., lower surface energy) substrates, such as hexamethyldisilazane (HMDS)-treated SiO_2_. When the energy of growth surface is lowered, indeed, the interaction between picene molecules and the substrate is weakened, and the molecular cohesion strength tends to prevail, enhancing the 3D character of the growth mode.^30^ It should be also remembered that the use of alternative dielectrics such as parylene was found to be very effective in reducing the occurrence of hysteresis and bias-stress phenomena which manifest strongly for SiO_2_-based picene devices.^32^ More recently, low voltage (|*V*_D_| < 6 V) flexible picene transistors were achieved by using ultrathin gate dielectrics based on aluminum oxide and monolayers of octadecylphosphonic acids on PEN (poly(ethylene 2,6-naphthalate)) substrates.^33^

Despite this intense work, the morphological and electrical properties of picene films were never investigated when this molecule was combined with other conjugated compounds to form heterostructures. Here, we report the fabrication and characterization of organic field-effect transistors bearing double-layer, triple-layer, and blended active channels achieved by the vacuum deposition of picene and *N*,*N*′-1*H*,1*H*-perfluorobutyl-1,6-dicyanoperylene-3,4:9,10-bis(dicarboximide) (PDIF-CN_2_) films.

Although the search for n-type organic compounds for high-mobility and air-stable transistors continues to progress, as demonstrated by recent publications,^34,35^ PDIF-CN_2_ remains a well-known and commercially available n-type organic semiconductor combining excellent self-assembling properties and remarkable stability in air.^36,37^ Thanks to the presence of cyano (−C≡N) groups in the bay region and of the fluoroalkyl (−CH_2_C_3_F_7_) side chains, the PDIF-CN_2_ surface is highly hydrophobic, and the transistor bearing PDIF-CN_2_ active channels can work even in a liquid environment.^38^ In the recent past, PDIF-CN_2_ was also used, in combination with rubrene, to study the intriguing behavior of single-crystal organic heterojunctions.^39^

In this work, a balanced ambipolar field-effect response in air was obtained with picene/PDIF-CN_2_ heterostructures through the proper selection of the transistor configuration and of the sequence of the fabrication steps. In such a way, the remarkable self-assembling features of both compounds can be preserved, and the response of the final devices is optimized by carefully tailoring the thickness of the various layers.

## Experimental Methods

For the transistor fabrication (bottom-gate configuration), commercial substrates, consisting of a 500 μm thick and highly doped silicon (Si^2+^) acting as a gate electrode and a 200 nm thick SiO_2_ dielectric barriers, were utilized. Before the organic film evaporation, all SiO_2_/Si^2+^ substrates were cleaned and functionalized by applying HMDS (hexamethyldisilazane) self-assembling monolayers by using a process lasting 7 days.^38^ In this way, the final water contact angle (θ_C_) of the SiO_2_ surface was increased up to about 110° (starting from an initial θ_C_ ∼ 60°).

**Figure 1:**
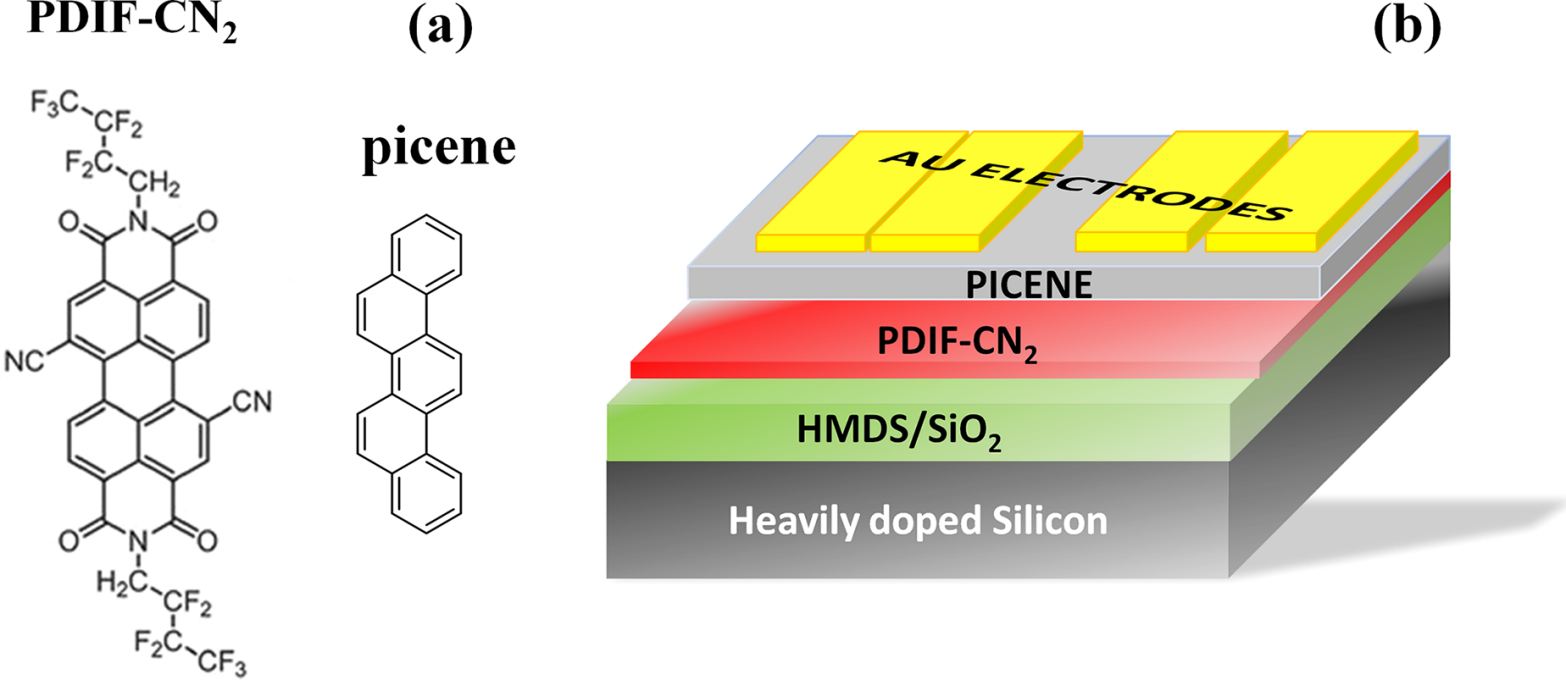
Sketch of the PDIF-CN_2_ and picene molecular structures (a). The bottom-gate top-contact picene/PDIF-CN_2_ device configuration mainly analyzed in this work (b).

Evaporated thin films based on picene and PDIF-CN_2_ molecules were employed as active layers of organic field-effect transistors (OFET). Molecular structures of picene and PDIF-CN_2_ are shown in Figure 1a, while a typical double-layer OFET structure with top-contact configuration is sketched in Figure 1b. All the investigated OFET devices were realized by the growth, under a vacuum of 10^–7^ mbar, of the two materials on our test substrates (HMDS-treated SiO_2_/Si^2+^). Unless otherwise stated, picene films were grown keeping the underlying substrate at room temperature and with a deposition rate (*R*) around 0.8 nm/min,^40^ while PDIF-CN_2_ films were deposited with heated substrates at about 110 °C and *R* ∼ 0.3 nm/min.^37^

Evaporated gold electrodes were used as source and drain contacts. Top-contact transistors were fabricated by thermally depositing gold electrodes (at 10^–5^ mbar and a deposition rate of about 2 nm/s, 40 nm thick) on the top of the organic layers through a metallic shadow mask to define the length (*L*) and width (*W*) of the active channels. In this study, we considered devices with *W* = 500 μm and variable channel length *L* = 200, 150, 100, and 50 μm. Alternative configurations, referred to the electrode position with respect to the organic layer stacking, were also explored: interdigitated bottom contacts (150 nm thick gold electrodes patterned on the SiO_2_ surface)^41^ and middle contacts (gold pads are deposited on the first organic layer before the deposition of the second one).

All OFET characteristics were recorded at room temperature in dark and in air by using a probe station connected to a Keithley 4200-SCS semiconductor parameter analyzer; the FET characteristics were measured in two-terminal mode in controlled environmental conditions (i.e., temperature set at 22 °C and humidity between 45% and 55%). The transfer curves for both p- and n-channel devices were analyzed to determine mobility (μ) and threshold voltage (*V*_th_) values by using the general formula for the saturation regime:
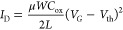
1where *I*_D_, *V*_G_, *V*_th_, *W*, *L*, and *C*_ox_ refer to drain current, gate voltage, threshold voltage, channel width, channel length, and capacitance per area of gate dielectric, respectively; the value of drain voltage (*V*_D_ = +50 for n-type, *V*_D_ = −50 for p-type) was fixed in the transfer curve measurement, while *C*_ox_ = 17.3 nF/cm^2^. The condition for a saturation regime, *V*_D_ > *V*_G_ – *V*_th_, was satisfied in the analysis of the transfer curve; in p-channel measurement mode, absolute values of *V*_D_, *V*_G_, and *V*_th_ (|*V*_D_|, |*V*_G_|, and |*V*_th_|) are employed for the analysis. A number of devices from a minimum of four to a maximum of eight per each channel length, thickness of the layers, and contact configuration were tested.

Film surface topographies and surface potential maps were acquired at a resolution of 512 × 512 pixels by noncontact atomic force microscopy (AFM) and amplitude-modulation scanning Kelvin probe microscopy (SKPM) techniques, respectively, by using a Park system Xe-100 microscope. AFM measurements were performed with a PPP-NCHR cantilever by NanoSensors (mechanical resonance at 300 kHz) while SKPM with Cr/Au-coated conducting cantilevers NSC14 Cr/Au MikroMash with typical mechanical resonances at 170 kHz. SKPM maps were acquired line by line in dual frequency mode: that is, acquiring simultaneously both the height profile and the potential profile of the scanned surface. For each line, acquired signals were demodulated by means of an external Stanford Research System SR830 DSP lock-in amplifier by using a sinusoidal reference with a frequency of 17 kHz and a *V*_AC_ amplitude between 1 and 1.5 V with a typical scan frequency of 0.1 Hz per line.

## Results and Discussion

Because the main goal of this experimental study was to fabricate OFETs showing improved ambipolar response, a device configuration based on double-layer active channel was first considered. Accordingly, our strategy was driven by the well-established knowledge about the deposition of picene and PDIF-CN_2_ layers with optimized structural properties. Because picene is a rather volatile compound, it is commonly evaporated while the growth surface is kept at room temperature.^42^ Conversely, PDIF-CN_2_ has been widely demonstrated to exhibit the best charge transport properties when the substrate is heated at *T*_sub_ = 110 °C during the deposition.^37^ This diverse behavior imposed a severe limitation in the double-layer configurations which could be analyzed. Indeed, the possibility to realize PDIF-CN_2_/picene structures (with picene being the underlayer) was soon discarded since any attempt to deposit a PDIF-CN_2_ layer on the top of a picene film kept at 110 °C produced a rapid steam of the picene molecules. The final devices achieved in this way displayed only a n-type response with degraded mobility values. At the same time, PDIF-CN_2_/picene heterostructures fabricated with both layers grown at room temperature show only a p-type response (see the discussion below). Based on these preliminary results, our attention was focused on the fabrication and characterization of the alternative double-layer configuration, where the PDIF-CN_2_ layer was first evaporated on HMDS/SiO_2_ with *T*_sub_ = 110 °C, and the heterostructures were subsequently completed with the deposition of the picene films on the PDIF-CN_2_ underlayer maintained at room temperature. The morphological properties of the so-obtained organic films and the electrical response of the related double-layer OFET will be the subject of the following sections.

### Film Morphology Characterization

**Figure 2:**
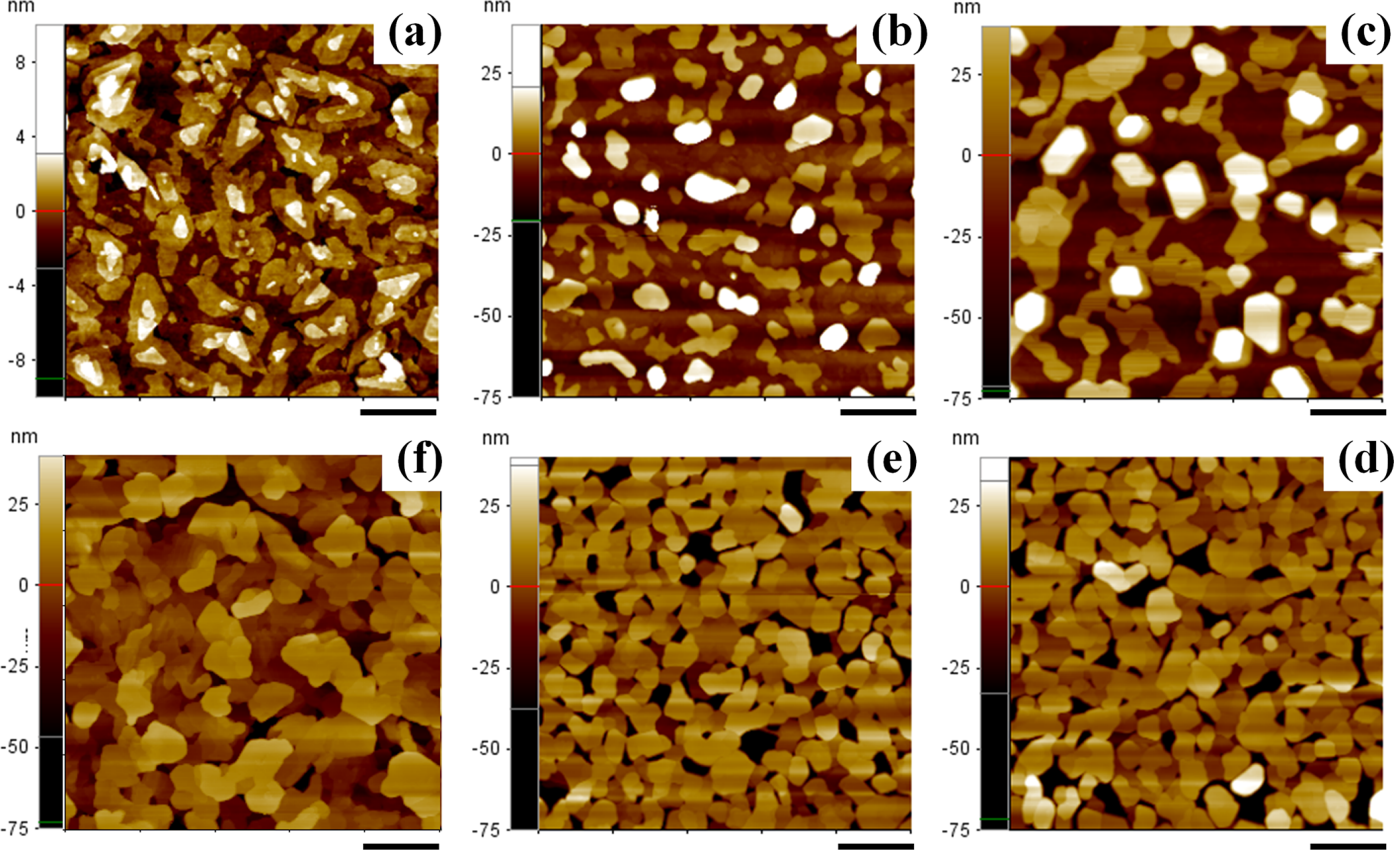
5 ×5 μm^2^ AFM images of the film surface: (a) 30 nm of PDIF-CN_2_, (b) picene/PDIF-CN_2_ heterostructure 5 nm/30 nm, (c) picene/PDIF-CN_2_ heterostructure 30 nm/30 nm, (d) picene/PDIF-CN_2_ heterostructure 60 nm/30 nm, (e) picene/PDIF-CN_2_ heterostructure 60 nm/15 nm, and (f) 60 nm of picene. The black sign is a reference of 1 μm.

AFM images in Figure 2 summarize the morphological properties of picene and PDIF-CN_2_ single layers (i.e., deposited on HMDS/SiO_2_) as well as those of various picene layers, with different thickness, grown on a PDIF-CN_2_ underlayer. Based on the previous discussion, picene was invariably evaporated keeping the growth surface at room temperature, while the PDIF-CN_2_ films were deposited on HMDS/SiO_2_, with *T*_sub_ = 110 °C. Figures 2a and 2f, in particular, report AFM images of the single layers as a reference for the typical morphologies of PDIF-CN_2_ and picene, respectively. As is well-known, when deposited in the optimized conditions, PDIF-CN_2_ films are composed of highly compact crystalline islands with a rounded shape (Figure 2a). Picene, on the other hand, displays a much more pronounced three-dimensional (3D) growth, and the related layers are characterized by well-identifiable columnar-shaped domains with a maximum diameter approaching 1 μm (Figure 2f). The surface roughness of single PDIF-CN_2_ films is typically lower than 2 nm, while the roughness of the picene layer is considerably larger due to the columnar-like film microstructure (see *w* values in Table 1).

**Table 1:** Morphological Parameters (Root-Mean-Square Roughness *w*, Surface Fractal Dimensionality α, the Recurrence Wavelength *λ*, and the Correlation Length *ξ*) Extracted by the HHCF Statistical Method (See Figure S2) from AFM Images of All the Thickness Combinations; the Picene/PDIF-CN_2_ Layer Thickness (in nm) Is Also Reported

sample	*w* (nm)	α	*λ* (nm)	*ξ* (nm)	*λ* – *ξ* (nm)
0/30	1.6	0.39	873	212	
15/30	10.6	0.56	647	196	451
30/30	36.3	0.55	999	379	620
60/30	16.7	0.51	635	167	468
0/15	1.2	0.54	433	88	
20/15	18.0	0.52	750	243	507
40/15	17.8	0.48	710	221	489
60/15	19.3	0.54	591	150	441
60/0	8.0	0.53	798	203	595

In Figures 2a–d, the series (0/30, 5/30, 30/30, and 60/30) of picene/PDIF-CN_2_ heterostructures (with the thickness of PDIF-CN_2_ underlayer fixed at 30 nm) is reported. In Figures 2d–f, conversely, the topography of the series (60/0, 60/15, and 60/30) of the picene/PDIF-CN_2_ bilayers is shown to highlight the effect of the underlayer thickness on the growth of 60 nm-picene film (60 nm is the thickness assuring the best electrical performances for our top-contact picene-based OFET). When analyzing Figures 2a–d at increasing picene thickness, it is possible to observe that already at a thickness of 5 nm the growth mode of picene on PDIF-CN_2_ is not layer-by-layer but mainly 3D. This type of growth, typically observed also on other surfaces,^30,32^ is here favored by the strong hydrophobicity of the PDIF-CN_2_ surface.^38^ In this case, the PDIF-CN_2_ surface covered by picene islands remains limited to about 30%. When the nominal thickness of picene is increased to 30 nm (always on 30 nm of PDIF-CN_2_), the coverage reaches the 50% threshold, even if the islands appear still weakly connected. Finally, the coverage degree rises up to 95% upon the deposition of 60 nm picene films. In this case (Figure 2e), a ripening effect for mounded structures can be observed providing a more compact film. The final size of the micrometric crystalline islands is, however, slightly reduced in comparison with that observable on the 60 nm thick picene single layer (Figure 2f) because of the increased surface roughness induced by the PDIF-CN_2_ layer respect to the SiO_2_ one (typical surface roughness of 0.2 nm). Significantly, (see Figures 2d–f) no evident differences in the picene film morphology evolution are observed when the thickness of the bottom PDIF-CN_2_ underlayer is reduced at 15 nm.

The crystalline quality of the PDIF-CN_2_, picene, and bilayers was checked by X-ray diffractometry, as shown in Figure S1. In the typical PDIF-CN_2_ film pattern (00*l*) diffraction peaks are mainly observed, indicating that the film islands are characterized by a preferred *c*-axis orientation (molecular long axis almost perpendicular to the growth surface). The picene films grown on the PDIF-CN_2_ result *c*-axis oriented, too (*c* = 13.5 ± 0.1 Å, very close to the single crystal value),^43^ exhibiting apparently a poor dependence on their nominal thickness or on the thickness of the PDIF-CN_2_ underlayer (see Figure S1).

To get more quantitative information about the film morphology and the growth mode, all the acquired AFM images were analyzed by the height–height correlation function (HHCF) statistical method (see Figure S2). In this way, we can extract the values of statistical parameters such as the heights distribution width (*w*, i.e., the root-mean-square roughness), the α parameter (related to the local fractal dimensionality of the surface), the recurrence wavelength (*λ*, related to the mean-space periodicity of the islands), and the correlation length (*ξ*, related to the mean dimension of the islands). They are all listed in Table 1. Here, the difference *λ* – *ξ* can be interpreted as a measure of the mean distance between the picene islands (in a certain way it is the measure of the degree of connection between the islands).^44^ Our attention is focused on this parameter because, generally, the charge carrier transport properties are remarkably correlated to the island contiguity as well as the quality of the grain boundaries.^45^ The minimum value of the *λ* – *ξ* difference is observed in the case of the heterostructure 60/15 (60 nm of picene grown on 15 nm of PDIF-CN_2_) which, in terms of morphological properties, represents the most promising choice for the ambipolar device realization. It should be also noticed that the α parameter is around 0.5 for all the picene films as a confirmation of the three-dimensional character of the related growth mode.^46^

### Ambipolar Response of Picene/PDIF-CN_2_ Hetrostructure OFETs

The electrical characterization of the samples investigated in this study started with the analysis of the single layer top-contact OFET. Figure S3 provides a general picture of the related electrical response, confirming the excellent quality of both picene and PDIF-CN_2_ films evaporated in optimized conditions on HMDS-treated SiO_2_/Si^2+^ substrate.

The output and the transfer curves reported respectively in Figures S3a and S3c (left panel) confirm the pure p-type response of the picene transistors (the presented data are referred to a device with the channel length *L* = 150 μm). Hence, the absolute drain current, |*I*_D_|, increases upon the application of a negative gate voltage (*V*_G_), and it is further enhanced by the progressive increase in |*V*_G_|. The output curves, |*I*_D_| vs |*V*_D_| plots, at different negative *V*_G_ values, provided typical normally off properties, indicating that the current flowing in the active channel is negligible when no gate voltage is applied. By analyzing devices on the same chip with different channel lengths (*L*), a marked dependence of the extracted field-effect mobility *μ*_p_ on *L* was observed. The highest *μ*_p_ value of the picene OFET is about 1.1 cm^2^ V^–1^ s^–1^ for *L* = 200 μm, while it is more than halved when *L* = 50 μm. This behavior is quite common for the OFET because of the so-called contact-resistance phenomenon which can be detected for both bottom- and top-contact devices.^47,48^ As typically found in previous reports, the value of the threshold voltage (*V*_th_) for picene devices on HMDS/SiO_2_ substrates is (in absolute value) large, being here close to −50 V and showing a poor dependence on *L*. For picene, this feature was commonly ascribed to a large density of charge trapping centers active at the interface between the organic semiconductor and the dielectric SiO_2_ surface.^29^

Single-layer PDIF-CN_2_ OFETs (Figure S3b and right panel in Figure S3c) exhibit coherently a n-type response, with the *I*_D_ enhancement being achieved through the application of positive *V*_G_. In very good agreement with literature,^36^ electron mobility (*μ*_n_) values extracted for this type of devices range between 0.2 and 0.3 cm^2^ V^–1^ s^–1^ with a much less pronounced (in comparison with picene) dependence on the channel length. This finding is clearly related to a minor impact of the contact resistance phenomenon. The threshold voltages, moreover, assumed small values which are typically comprised in the range between −5 and +5 V. This explains the usually observed capability of these OFETs to carry a non-negligible *I*_D_ current even when *V*_G_ = 0 V.^37^

**Figure 3:**
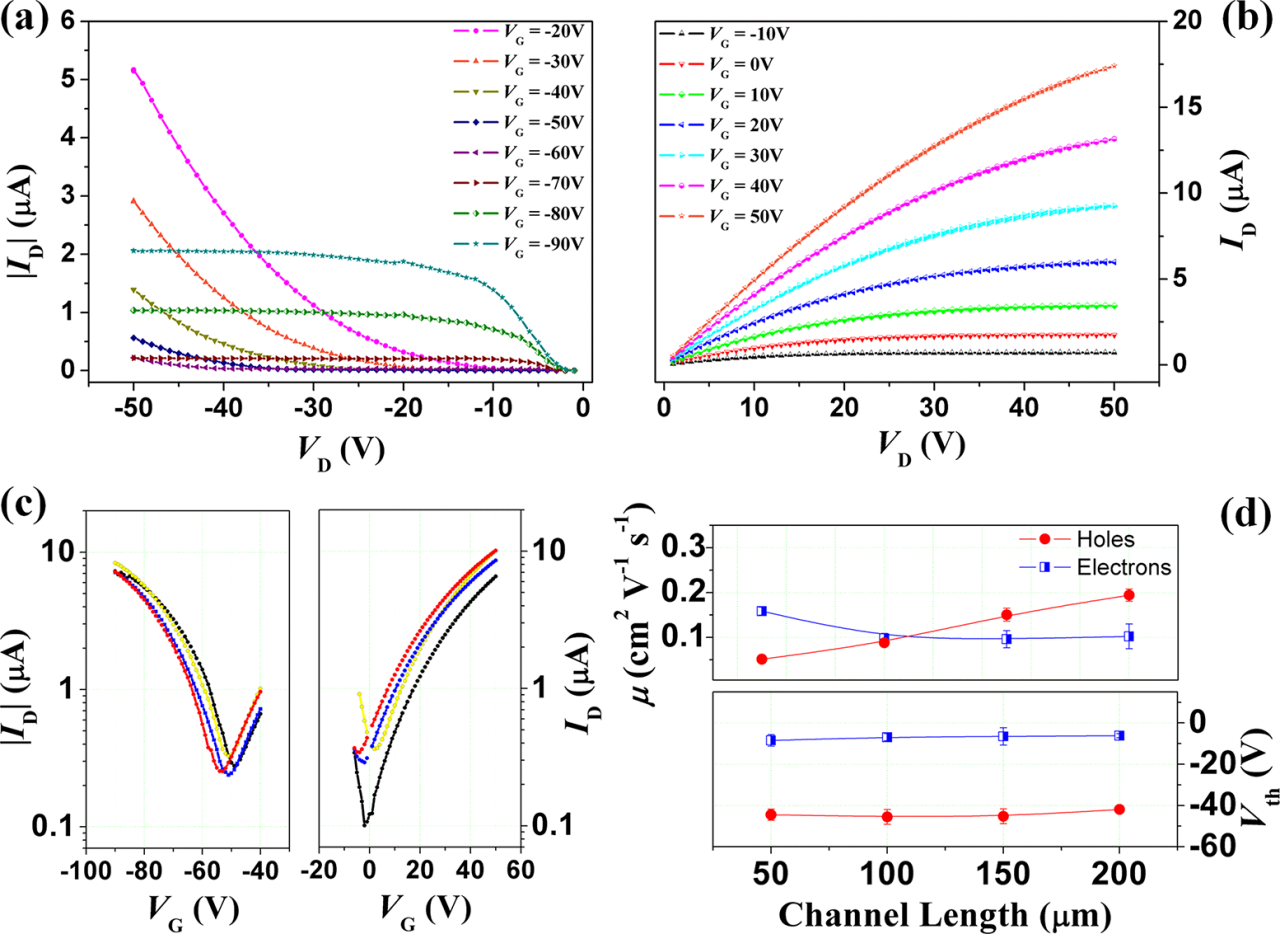
Output currents for p-type (a) and n-type (b) charge carriers transport for the heterostructure-based FET with thicknesses of PDIF-CN_2_ 15 nm and picene 60 nm are reported for different gate voltages. For better clarity, here only the output curves of a device with channel length of 150 μm are plotted. (c) Transfer curves of devices with channel length of 50, 100, 150, and 200 μm are shown for both the n and p branches (*V*_D_ = 50 V and *V*_D_ = −50, respectively). In (d) are plotted the mean mobility and threshold voltage values of the picene/PDIF-CN_2_ devices with the different channel lengths.

**Figure 4:**
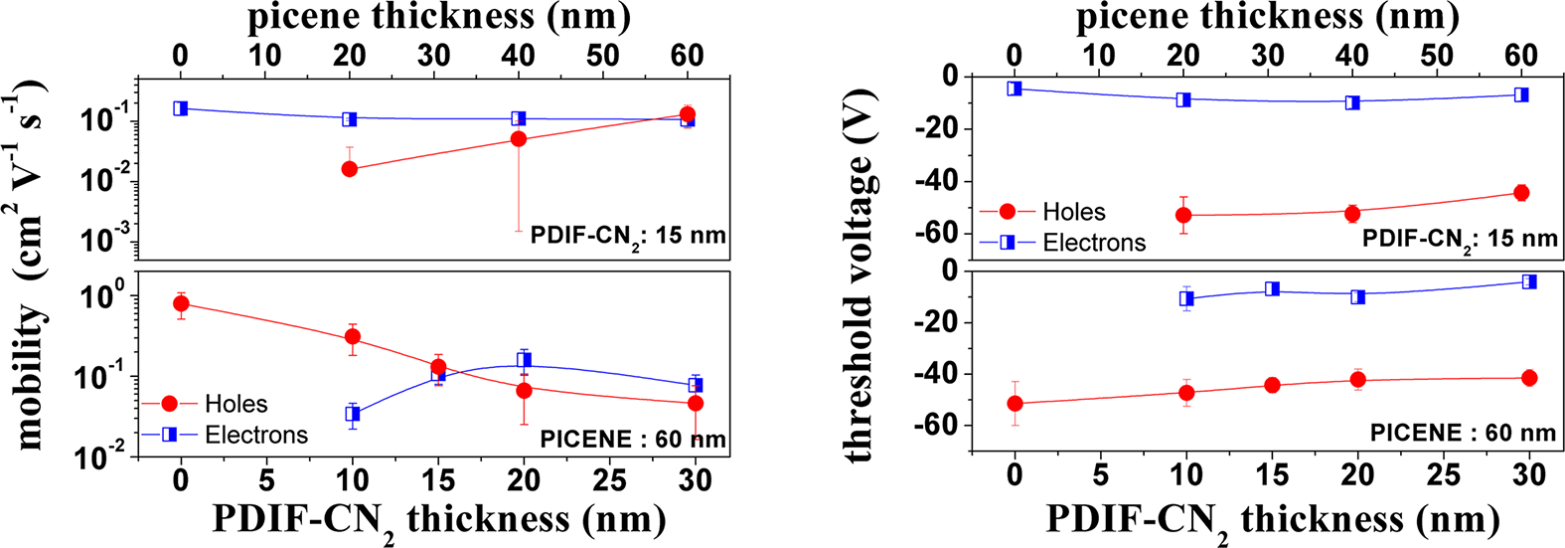
In the top panels is shown the behavior of the mean values of the p and n mobility and threshold voltages of the heterostructure-based devices, realized fixing PDIF-CN_2_ thickness at 15 nm and changing the picene upper-layer thickness from 60 to 0 nm (case 1). In the bottom panels is shown the behavior of the mean values of p and n mobility and threshold voltages of the heterostructure-based devices realized fixing the picene thickness at 60 nm and changing the PDIF-CN_2_ underlayer thickness from 0 to 30 nm (case 2). All the date are referred to devices with *L* = 150 μm.

Once assessed the single layer devices and confirmed the optimized electrical performances of the deposited films, our efforts were focused to analyze the response of double-layer OFETs based on the picene/PDIF-CN_2_ structures. The morphological analyses introduced in the previous section suggested that the coverage degree and morphological quality (i.e., the increase of coverage and connection between the islands) of picene films grown on PDIF-CN_2_ underlayer are optimized for a thickness of 60 nm. In Figures 3a–c, the output and transfer curves for a top-contact picene/PDIF-CN_2_ (respectively 60 and 15 nm thick) OFET with *L* = 150 μm are shown. A clear ambipolar response is observed for this device, providing the possibility to achieve the *I*_D_ enhancement for both positive and negative *V*_G_ voltages. This ambipolar character is also confirmed by the observation that in the p-type output curves the *I*_D_ behavior at low |*V*_G_| and high |*V*_D_| is dominated by the injection of electrons occurring at the drain electrode (i.e., in these conditions, the *V*_G_ – *V*_D_ voltage difference results largely positive and electrons can be accumulated in the semiconducting region near the drain contact). The dual effect (i.e., hole injection from the drain contact) cannot be observed in the n-type output curves (Figure 3b) because of the largely negative *V*_G_ values required to provide the hole accumulation regime. Figure 3d summarizes the average mobility and threshold voltage values estimated for this double-layer ambipolar device as a function of the channel length. Maximum hole mobility values, related to the picene active channel, are about 0.2 cm^2^ V^–1^ s^–1^, being considerably reduced in comparison with those estimated for the single-layer transistors. This feature can be associated with the smaller size of the crystalline picene islands (directly comparable in Figures 2e and 2f; see also the *ξ* parameter in Table 1). However, *μ*_p_ keeps its linearly decreasing behavior at reducing channel length. Significantly, at the same time, the threshold voltages are decreased (in absolute value), being about −40 V. The charge transport properties of the n-type PDIF-CN_2_ channel are much more similar to those observed for the related single-layer devices. Electron mobility remains larger than 0.1 cm^2^ V^–1^ s^–1^ with a weak dependence of the channel length. The threshold voltages are only slightly shifted toward more negative values (∼−5 V). As a whole, in particular for *L* = 150 and 100 μm, the ambipolar response of this device is rather balanced in terms of mobility for the p- and n-carriers. Figure 4 offers a synthetic view of all the experimental results achieved by fabricating and electrically characterizing various double-layer picene/PDIF-CN_2_ OFETs. These tests were conducted by systematically modifying the thickness of the two layers, with the goal to identify the combinations providing the best mobility performances.

Basically, we followed two routes. Case 1: the PDIF-CN_2_ underlayer thickness was fixed at 15 nm, and the picene thickness was increased from 5 to 60 nm, to analyze the device performances while increasing the picene coverage degree. For this set of samples, the mean μ and *V*_th_ parameters are summarized in the top panels of Figure 4. As shown, while the n-channel mobility remains rather constant, p-channel mobility raises up remarkably (by more than 1 order of magnitude) when the picene thickness goes from 20 to 60 nm (the sample with picene thickness of 5 nm did not display any p-type response). These findings are obviously related to the typical 3D growth mode of picene and confirms the results of the morphological analysis, suggesting that a thickness of 60 nm is required for picene films to obtain well-connected islands and better charge transport properties. Case 2: picene thickness was fixed at the optimum value of 60 nm, while changing the thickness of the PDIF-CN_2_ underlayer from 10 to 30 nm. Mean values of *μ* and *V*_th_ are summarized in Figure 4 (see bottom panels). In this way, it was observed that by increasing the PDIF-CN_2_ thickness, the hole mobility related to the picene channel is monotonously decreasing. Conversely, electron mobility is enhanced by increasing the PDIF-CN_2_ thickness and tends to saturate when the layer thickness overcomes the size of the field-effect charge accumulation region (Fermi length is about 6 nm).^49^ Following this second route, it is again confirmed that the best p- and n-charge mobility (*μ* ∼ 0.15 cm^2^ V^–1^ s^–1^) balancing is achieved for the double-layer heterostructure with 15 nm of PDIF-CN_2_ and 60 nm of picene. The panels in Figure 4 also show a slight shift of *V*_th_ for the n-type response toward more negative values (between −5 and −10 V), which should be related to the presence of a low-density charge accumulation region at the picene/PDIF-CN_2_ interface (see the results of the SKPM analysis below). On the other hand, in the presence of the PDIF-CN_2_ underlayer, the *V*_th_ values for the p-type response decreases (in absolute value), approaching −40 V. This trend is further favored by the increased thickness of PDIF-CN_2_. Such behavior might also be put in perspective considering the PDIF-CN_2_ film as an additional buffer layer which might favor the electrochemical stability of p-type transport.

In conclusion of this section, it should be also remarked that the position of the gold electrode plays a fundamental role in determining the final response of these double-layer picene/PDIF-CN_2_ OFET. Even considering the optimized combinations of PDIF-CN_2_ and picene thicknesses, devices having electrodes deposited before the growth of the two organic layers (bottom-contact) or between the PDIF-CN_2_ and the picene layers (middle-contact) exhibit only a n-type response (see Figure S4, referred to a middle-contact sample). Basically, this result is coherent with previous studies highlighting the difficulty to effectively inject and collect charges in and from respectively a picene layer when this is evaporated on prefabricated electrodes.^30^

### PDIF-CN_2_/Picene/PDIF-CN_2_ Triple-Layer and Picene/PDIF-CN_2_ Blend OFETs

**Figure 5:**
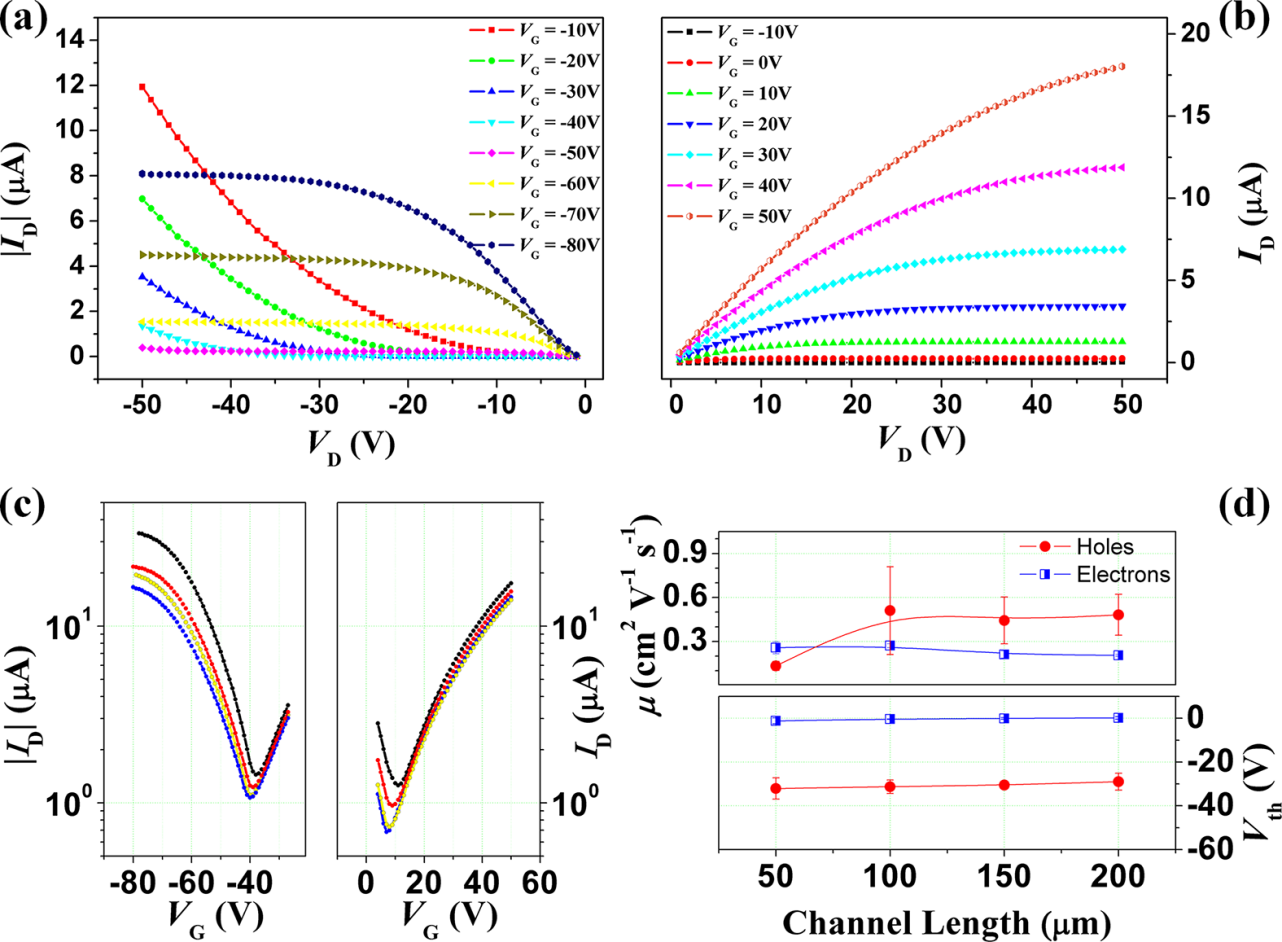
Output currents for p-type (a) and n-type (b) charge carriers transport for the trilayer-based FET with the sequence PDIF-CN_2_ (15 nm)/picene (60 nm)/PDIF-CN_2_ (5 nm) are reported for different gate voltages. For better clarity, here only the output curves of a device with channel length of 150 μm are plotted. (c) Transfer curves of devices with channel length of 50, 100, 150, and 200 μm are shown for both the n and p branches acquired at *V*_D_ = 50 V and *V*_D_ = −50, respectively. In (d) are plotted the main mobility and threshold voltage values of the PDIF-CN_2_/picene/PDIF-CN_2_ devices with the different channel lengths.

In analyzing the response of various double-layer OFET configurations, we also assessed the behavior of PDIF-CN_2_/picene OFETs, where the PDIF-CN_2_ layer was evaporated on the picene underlayer, kept at room temperature. As known, when deposited by the Knudsen cell with *T*_sub_ = room temperature, the morphological quality of PDIF-CN_2_ films is very poor, and the layers are basically composed of small rounded grains.^50^ These features were confirmed here when PDIF-CN_2_ was evaporated, with different thickness (3 and 15 nm), on a 60 nm thick picene underlayer (see the X-ray diffraction pattern in Figure S1 and AFM images in Figure S5). Accordingly, top-contact devices based on this double-layer structure showed only a p-type response, being the room-temperature-grown PDIF-CN_2_ film unable to effectively transport electrical current (Figure S6). Interestingly, however, this top layer affects the overall performances of the picene active channel. Although, at increasing PDIF-CN_2_ thickness, hole mobility slightly decreases in comparison with the picene single-layer devices, we also observed a considerable decrease (in absolute values) of the threshold voltages being shifted toward −30 V (the typical values for single-layer picene OFET range between −50 and −60 V). This occurrence was achieved already with a very thin (nominally 3 nm) PDIF-CN_2_ layer, suggesting the interfacial nature of this phenomenon and the ability of the layer to completely cover the picene surface. This observation, related to the insertion of a thin PDIF-CN_2_ layer between picene film and the gold electrodes, confirms the relevance of the detailed chemical and structural nature of the injecting contacts for device performance optimization.^51^ Moreover, this effect is qualitatively similar to what observed in previous experiments when a thin layer of the fluorinate small molecule 2,3,5,6-tetrafluoro-7,7,8,8-tetracyanoquinodimethane (F4-TCNQ) was utilized in the same position for an equivalent picene thin-film transistor.^52^ So the observed *V*_th_ shift in the picene transfer curves should be related to a hole doping effect induced by the strong electron-acceptor character of the PDIF-CN_2_ compound, as recently found also for other p-type compounds when combined even with fluorinated self-assembled monolayers.^53^ Inspired by the aforementioned results, a triple-layer heterostructure was fabricated by sequential deposition of 15 nm of PDIF-CN_2_ (grown at *T*_sub_ = 110 °C) as bottom layer, 60 nm of picene as middle layer, and 5 nm of room-temperature-grown PDIF-CN_2_ as top layer. The device was then completed by the evaporation of gold source–drain contacts (top-contact configuration). Figures 5a–c show the transfer and output characteristics of a PDIF-CN_2_/picene/PDIF-CN_2_ OFET with *W* = 500 μm and *L* = 150 μm. Clear ambipolar behavior is again observed in the *I*_D_ vs *V*_G_ plots. The dependence of *μ*_p_ and *μ*_n_ on the channel length estimated for this type of triple-layer device is shown in Figure 5d. Different from the corresponding double-layer heterostructure, the hole (*μ*_p_) mobility remains here quite constant, except for *L* = 50 μm, with the maximum value of about 0.5 cm^2^ V^–1^ s^–1^ at *L* from 100 to 200 μm. Coherently with previously discussed results, the electron *μ*_n_ mobility value does not vary against *L* and assumes values very close to 0.2 cm^2^ V^–1^ s^–1^. In agreement with the observations discussed at the beginning of this section, it was confirmed the large shift of the threshold voltages for the p-type response which, even in this case, are approximately equal to −30 V.

For the sake of completeness, a few devices were fabricated by using also a coevaporation process for the active channel definition. In this case, the HMDS/SiO_2_ substrates were kept at room temperature, and the deposition rate chosen for picene was considerably larger (i.e., a 6× factor as for the device in Figure S7) than that adopted for PDIF-CN_2_. This choice was motivated by the awareness of the disordered character of the PDIF-CN_2_ evaporated in these conditions, while picene can preserve its self-assembling properties. The AFM image in Figure S7a shows the morphology of the picene/PDIF-CN_2_ blend, revealing a considerable decrease of the size of the islands in comparison with that observed for the single-layer picene that results crystalline and *c*-axis oriented as deduced by X-ray diffraction pattern in Figure S1i. The electrical response of the OFET bearing coevaporated active channels displayed only a p-type response (Figure S7b), with the above-discussed morphological features directly translated in a reduction of hole mobility (*μ*_p_) values down to 0.1 cm^2^ V^–1^ s^–1^ for all the channel lengths. The observation that even in this case the threshold voltage values are in the range between −30 and −40 V seems to suggest that the direct interaction between picene and PDIF-CN_2_ molecules or nanoclusters (and not only between compact layers) can improve the stability of the threshold voltage and to reduce associated trapping effects. In a very characteristic way, PDIF-CN_2_ could be used both as electron-transporting layer and hole-doping compound as a function of the deposition conditions (namely, the different temperature of the growth surface).

### SKPM Analysis of the Picene/PDIF-CN_2_ Heterojunction

Scanning Kelvin probe microscopy (SKPM) is a powerful technique,^54^ first introduced to measure the work function of metals and more recently applied for the quantitative analysis of contact resistances in coplanar OFETs,^47,49,55^ charge dynamics,^56^ or to reveal important information about interface charge,^57^ charge transfer,^58^ and charge trapping^59^ at domain interfaces.

With this technique, a conductive tip scans the sample surface, and the difference between their energy vacuum levels results in a contact potential difference (CPD); an electrostatic force between tip and sample is added to the atomic one. The SKPM measurement consists in the pointwise nullification of this electrostatic force contribution by applying an external potential (*V*_ext_ to the tip in our case) which nullifies the CPD, acquiring concomitantly the morphology of the scanned area.

**Figure 6:**
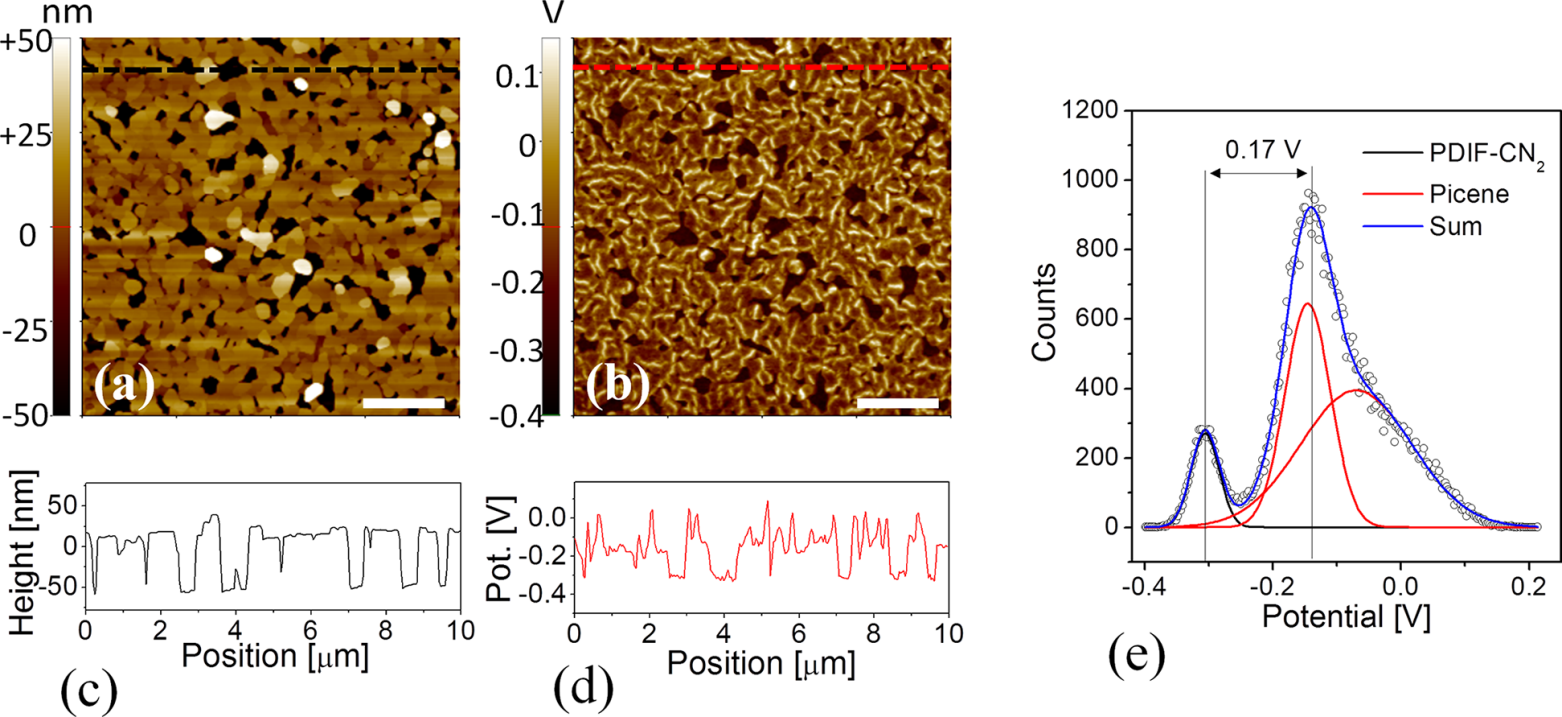
(a) 10 × 10 μm^2^ topography of a picene/PDIF-CN_2_ (60 nm/15 nm) heterostructure and (c) corresponding height line profile (black dashed line in (a)). (b) Surface potential mapping of (a) acquired via SKPM and (d) corresponding potential profile (red dashed line). The white marker is 2 μm. (e) Statistical distribution of surface potential discerned from (b) highlighting the presence of multiple peaks corresponding to the two organic materials. A multipeak fit of the histogram is plotted (blue line) as sum of contributing Gaussian distributions (red lines from picene and black line from PDIF-CN_2_).

A typical example of SKPM image on picene/PDIF-CN_2_ heterostructure is shown in Figure 6, where a 10 × 10 μm^2^ topography and the corresponding surface potential map are reported. The picene thin film (60 nm in this case) evaporated on the PDIF-CN_2_ (15 nm) substrate is characterized by flat-terminated cylindrical pillars 80–100 nm high (Figure 6c). For the reported surface, a picene coverage of about 92% is estimated. The surface potential of the heterostructure retraces the morphological features of picene grains which are observed to be characterized by higher potential values (less negative) respect to the PDIF-CN_2_ exposed surface (Figure 6d). Two statistical distributions of the surface potential, one for each layer, are obtained as shown in Figure 6e. Notably, while the PDIF-CN_2_ contribution can be identified as a peak at lower potential and composed by a single Gaussian curve, the peaked curve relative to the picene is typically formed by the convolution of two distinct distributions. In particular, the one at higher voltages is related to the brighter wormlike areas in the potential map (Figure 6e). These localized potential areas could be related to some positive charges or strain localized in structural defects inside the picene islands, presumably dislocations or internal grain boundaries.^60−62^ No effects are observed reducing the coverage of picene islands on PDIF-CN_2_, confirming that the measure is unaffected by crosstalking or artifacts (Figure S8).

**Figure 7:**
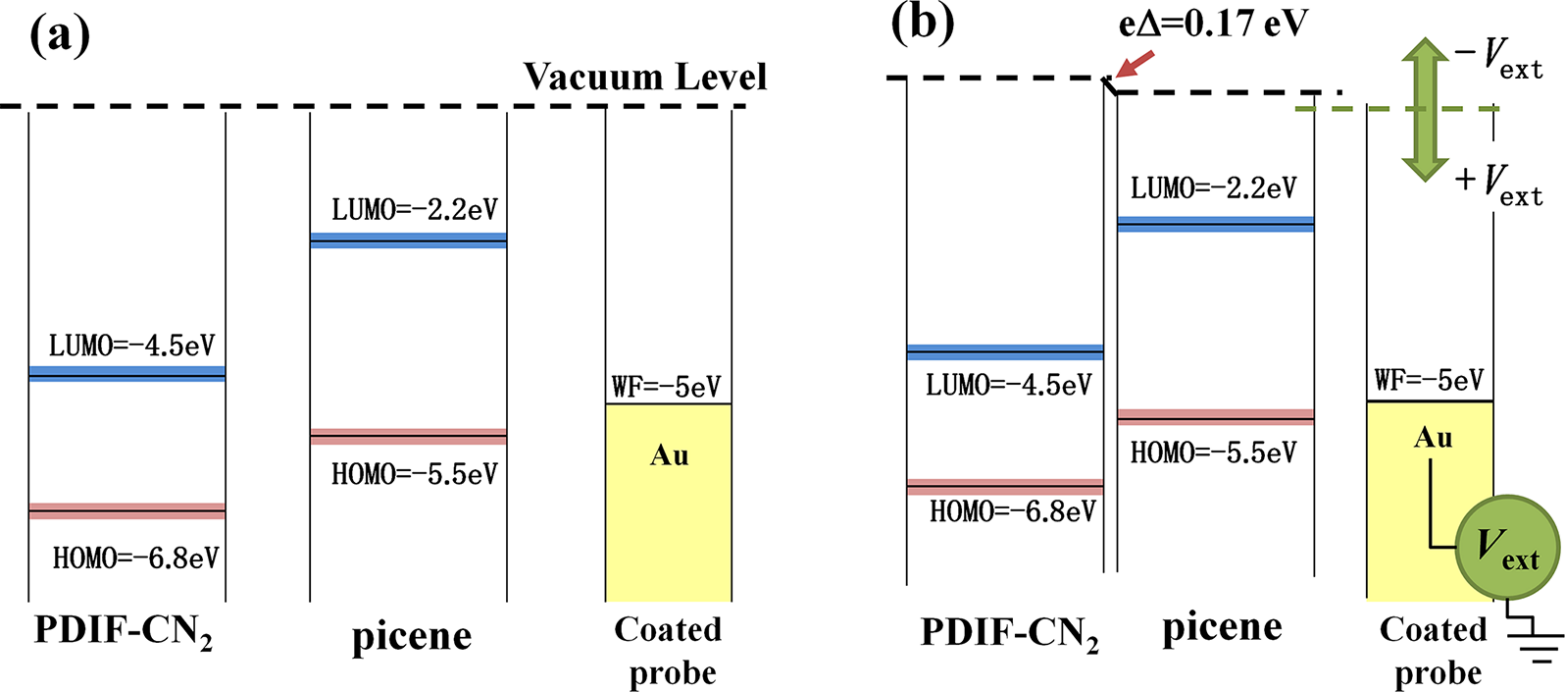
(a) Energy band and molecular level diagram for isolated materials and out of contact gold coated AFM tip. (b) Energy band and molecular level diagram for the picene/PDIF-CN_2_ heterostructure.

By fitting the statistical distribution of Figure 6e, a peak-to-peak potential difference, Δ = 170 mV (with a tolerance of 20 mV), is estimated between the PDIF-CN_2_ and the picene thin films. In particular, when picene/PDIF-CN_2_ and the gold-coated tip are in contact the Δ value between the layers represents the difference between the vacuum levels of the single films for an electron,^63,64^ as illustrated in Figure 7, where the heterojunction energy level diagram (type II staggered gap) is sketched. Notably, the vacuum level of PDIF-CN_2_ is higher than that of picene, suggesting the interface dipole in picene/PDIF-CN_2_ points from picene to PDIF-CN_2_.

**Figure 8:**
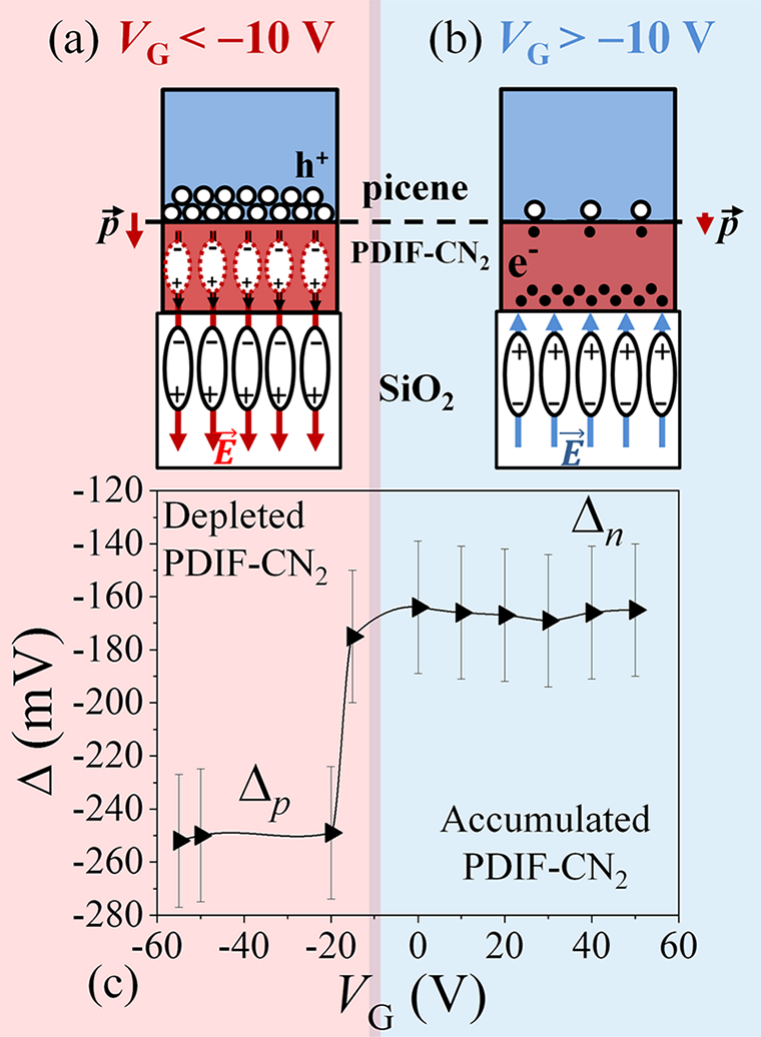
(a) Schematic depiction of the double interface picene/PDIF-CN_2_/SiO_2_ for *V*_G_ < −10 V. In this case, the charge depleted PDIF-CN_2_ layer can be considered as a plain dielectric interface which allow holes to be accumulated at the picene/PDIF-CN_2_ interface. (b) The same scheme for *V*_G_ > −10 V and fully accumulated PDIF-CN_2_ layer highlighted by the presence of electrons at the PDIF-CN_2_/SiO_2_ interface. (c) Potential difference Δ measured by SKPM as a function of the externally applied gate–source bias (*V*_G_).

Further considerations can be derived from the analysis of Δ as a function of the applied gate bias as reported in Figure 8. Varying the externally applied gate voltage between −55 V < *V*_G_ < +50 V, two different states are observed according to the n-type (p-type) kind of behavior of the single organic layer. For *V*_G_ > −10 V, charge carriers are accumulated at the PDIF-CN_2_/SiO_2_ interface, allowing n-type transport. In this configuration, the accumulation layer acts as an electrostatic shield for the overimposed picene thin film which is thus not influenced by the external gate field (Figure 8b). As a consequence, Δ_n_ is observed to be independent from *V*_G_, settling again around 170 mV (Figure 8c). Conversely, for *V*_G_ < −10 V, the PDIF-CN_2_ channel is depleted from electrons. In such a condition, the PDIF-CN_2_ thin film starts to act as an additional dielectric layer (Figure 8a), allowing the gate electric field to penetrate and consequently inducing holes accumulation at the picene/PDIF-CN_2_ interface. The Δ_p_ contribution decrease its value to −250 mV as deduced from Figure 8c.

The magnitude and sign of the Δ value and the related dipole bring us to the conclusion that at the picene/PDIF-CN_2_ interface charge transfer is negligible; space charge is accumulated with low density, and weak band bending occurs. These interface properties do not affect the field-effect device standard working mode and make manageable the engineering of heterostructured OFETs for the ambipolar charge transport.

## Conclusions

In this study, we fabricated and characterized the response of various OFET heterostructures featuring double-layer, triple-layer, and codeposited active channels, achieved through the evaporation of picene and PDIF-CN_2_ molecules. Our main goal was to identify the configuration able to provide the best performances in terms of balanced ambipolar response (i.e., capability to accumulate both holes and electrons as a function of the applied *V*_G_ voltages).

The experimental data here discussed confirm that picene is characterized by a predominant 3D growth and rather thick (e.g., >50 nm) films should be grown to guarantee the proper structural connectivity of the crystalline islands and, consequently, good charge transport properties. When evaporated on heated surfaces, on the other hand, PDIF-CN_2_ displays excellent and reliable self-assembling properties even for thin (∼10 nm) films. For both these compounds, the correlation between morphological and electrical properties results is straightforward.

In reason for this feature, a good ambipolar response featuring balanced mobility values of ∼0.1 cm^2^ V^–1^ s^–1^ can be achieved in double-layer OFET with a PDIF-CN_2_ underlayer deposited with *T*_sub_ = 110 °C (thickness between 15 and 30 nm) and a 60 nm thick picene overlayer, evaporated by keeping the growth surface at room temperature. In this configuration, the use of top-contact gold electrodes is mandatory to achieve an effective injection for both holes and electrons. This requirement is related to the need to attenuate the contact resistance effect being particularly severe for picene active channels. We also found that room-temperature-grown PDIF-CN_2_ layers (a few nanometers) can be inserted between picene channels and the gold electrodes, preserving the high mobility values but remarkably reducing (in absolute value) the threshold voltages of the p-type response related to the hole doping effect induced by the strong electron-acceptor character of the PDIF-CN_2_. This observation has been exploited to fabricate a triple-layer OFET heterostructure, exhibiting improved performances in comparison with the double-layer configuration. A consistent reduction of the threshold voltages has been observed also in p-type devices based on codeposited active channels, where PDIF-CN_2_ was evaporated at a much lower rate than picene. Finally, scanning Kelvin probe microscopy performed on picene/PDIF-CN_2_ heterojunctions gave indications about the formation of a space charge accumulation layer with low density at the interface between the two compounds.

As a whole, the findings here reported suggest that when grown on PDIF-CN_2_, the hole-transporting properties of picene films are more robust versus the charge trapping effects which tend to considerably affect the absolute values of the related threshold voltages. According to our analysis, this phenomenon should be associated with a reduction of the density of the residual water molecules absorbed on the growth surface (i.e., PDIF-CN_2_ is more hydrophobic than SiO_2_) rather than morphological/structural defects in picene layers. In the same direction, when triple-layer heterostructures are taken into account, the mechanical effect of the PDIF-CN_2_ fluorinated side chains, acting as a capping layer which hampers the penetration of environmental gases,^65^ should play an additional and beneficial role. Finally, the formation of interface charges between PDIF-CN_2_ and picene molecular domains, although with low density, contribute positively to the improvement of the charge transport performances in the analyzed devices.

## Supporting Information Available

X-ray diffractogram of picene/PDIF-CN_2_ heterostructures, single layers and blend film; fitting curves of the height–height correlation data extracted from the AFM images in Figure 2; electrical characterization of picene (60 nm thick) and PDIF-CN_2_ (15 nm thick) single layers based OFETs; electrical characterization of middle-contact bottom-gate devices; AFM images and electrical characterization of inverted PDIF-CN_2_/picene heterostructure; AFM image and electrical characterization of picene/PDIF-CN_2_ blend; AFM image and potential maps of picene/PDIF-CN_2_ heterostructure a different coverage (PDF)
