# Supplementary material for: Balanced Ambipolar Charge Transport in Phenacene/Perylene Heterojunction-Based Organic Field-Effect Transistors
Source: ACS Appl Mater Interfaces. 2021 Feb 15;13(7):8631–42. doi: 10.1021/acsami.0c20140 (PMC9289882; doi:10.1021/acsami.0c20140)
Supplement: Supplementary file 1 — am0c20140_si_001.pdf [file am0c20140_si_001.pdf]

Supporting Information

Balanced ambipolar charge transport in phenacene/perylene heterojunction-based organic field-effect transistors

Tomoya Taguchi[1], *Fabio Chiarella*[2,*], *Mario Barra*[2], *Federico Chianese*[3,2], *Yoshihiro Kubozono*[1], *Antonio Cassinese*[3,2]

[1] Research Institute for Interdisciplinary Science, Okayama University, Okayama 700-8530, Japan.

[2] CNR-SPIN, c/o Dip. di Fisica “Ettore Pancini”, P.le Tecchio, 80, I-80125 Napoli, Italy.

[3] Dip. di Fisica “Ettore Pancini”, Università ‘Federico II’, P.le Tecchio, 80, I-80125 Napoli, Italy.

* Corresponding author: fabio.chiarella@spin.cnr.it

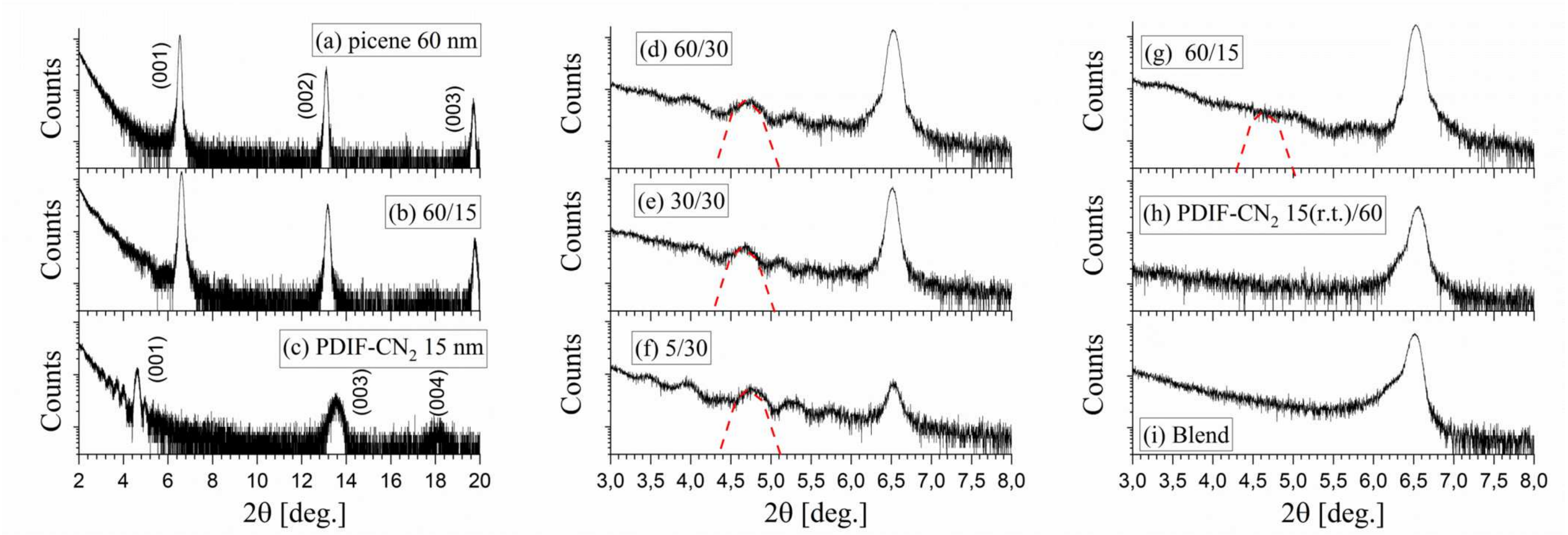

Figure S1 X-ray diffraction pattern in of (a) picene and (c) PDIF-CN$_2$ single layer (as reference), (b) picene/PDIF-CN$_2$ bilayer with thicknesses of 60 nm / 15 nm, respectively. In (d). (e) and (f) graphs are plotted the spectra of the series of bilayer at fixed PDIF-CN$_2$ thickness (30 nm) and with different picene thickness (60 nm, 30 nm and 5 nm, respectively). In (g) is reported the picene (60 nm)/PDIF-CN$_2$ (15 nm) bilayer spectra while in (h) is presented the inverted structure bilayer with room-temperature-grown PDIF-CN$_2$ on the top of the 60 nm thick picene film. (i) X-ray diffraction pattern of the co-evaporate picene/PDIF-CN$_2$ blend film. Measurements are acquired using a Rigaku diffractometer in Bragg-Brentano configuration and CuKα emission wavelength. The presence of the PDIF-CN$_2$ peak is glimpsed (red dashed curves) because of the picene thickness.

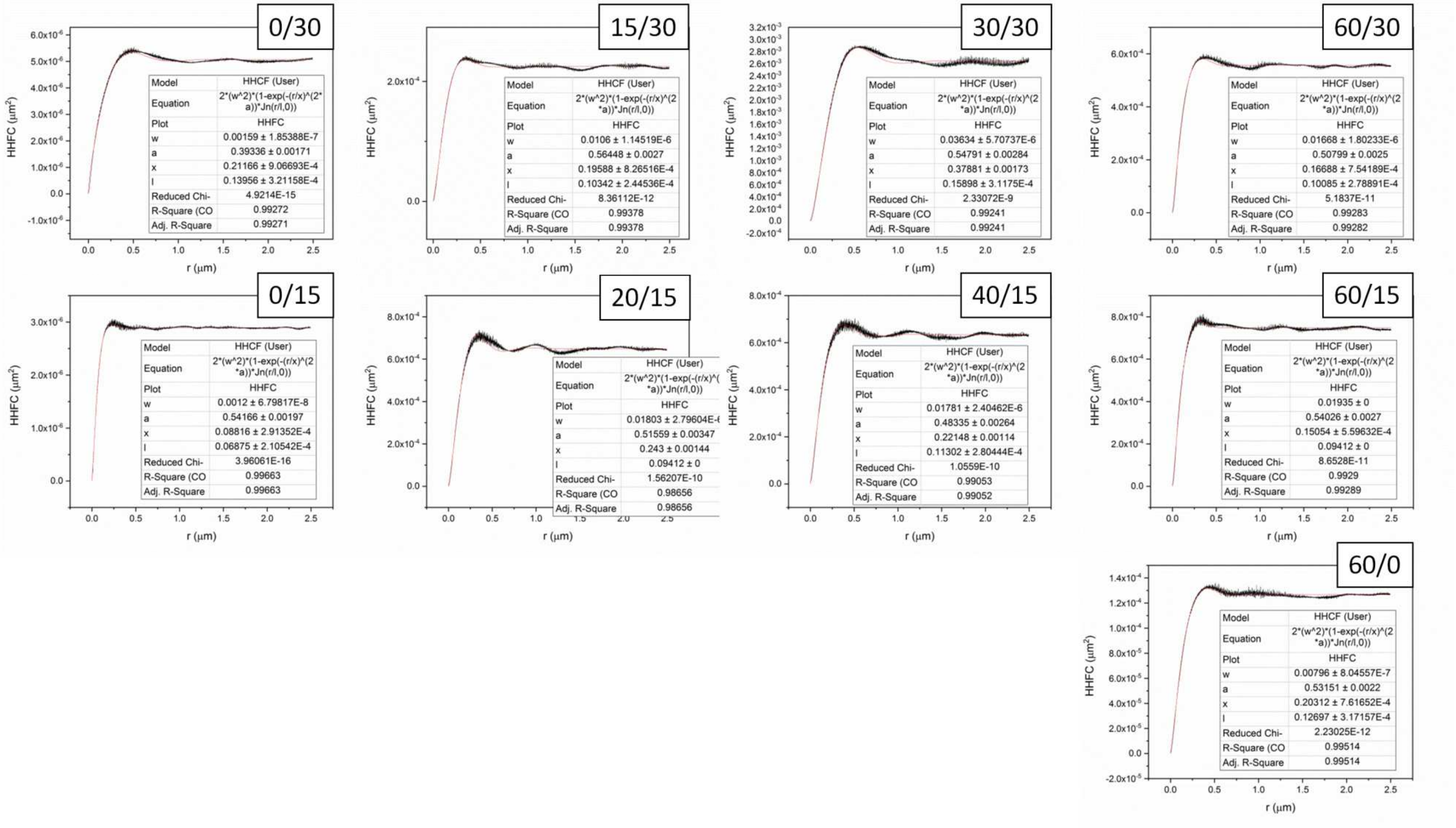

Figure S2 Height-height correlation data (in black) extracted from the AFM images of Figure 2 in the main text (the numbers at the top of each

graph represent the thicknesses of the picene and PDIF-CN_2 layers, respectively). The fitting curves (in red) are made following the height-height correlation function definition for mounted morphologies:

$$H(r) = 2w^2[1 - exp[-(r/\xi)^{2\alpha}] \cdot J_o(2\pi r/\lambda)].$$

For more details see ref.[1]:

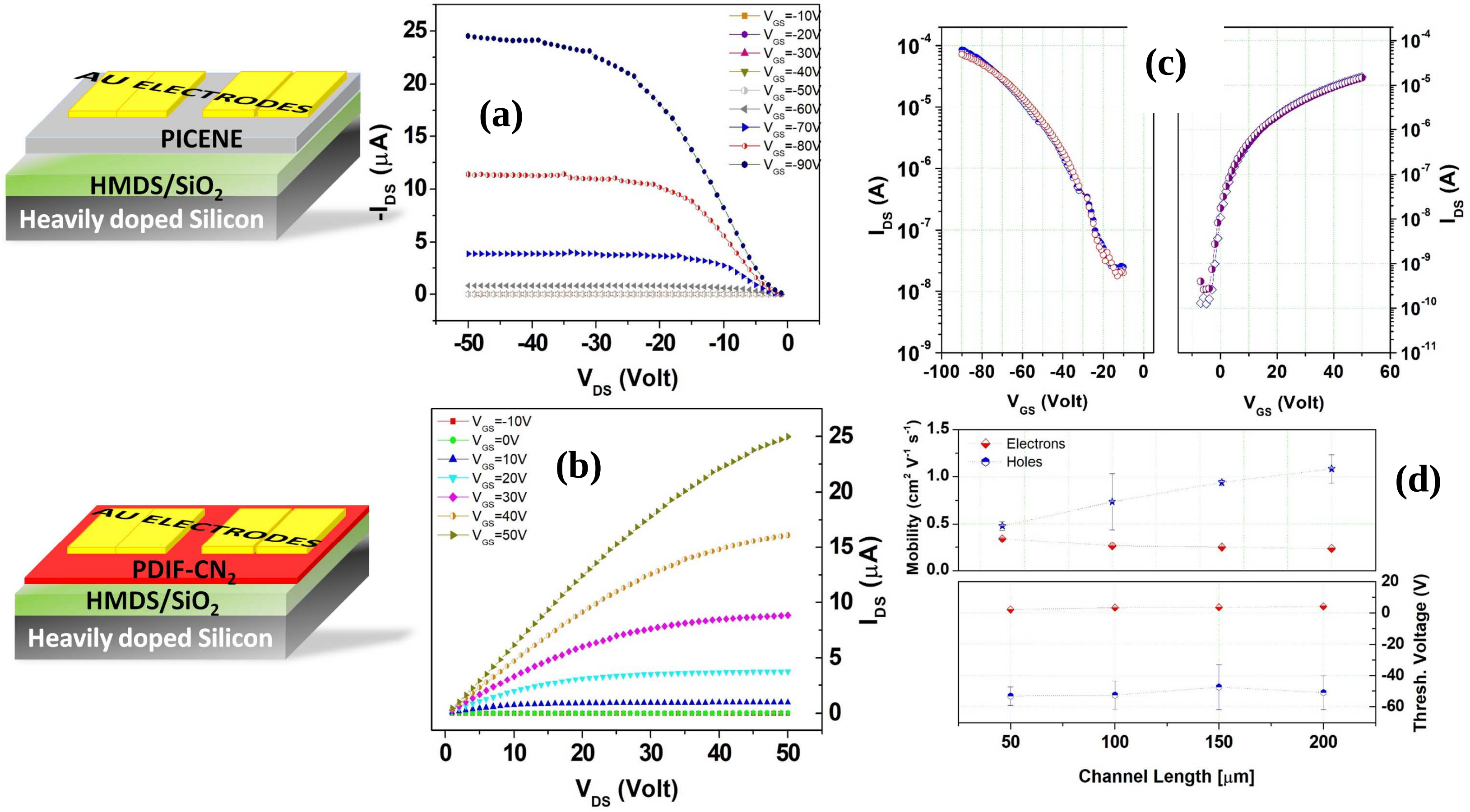

Figure S3 Electrical output characteristics of picene (60 nm thick) (a) and PDIF-CN_2 (15 nm thick) (b) single layers based devices for transistor width L=150 μm are plotted. to the corresponding device sketches, the output curves, measured, are presented. Transfer curves in saturation regime (c) and the extracted mobility and threshold voltage vs. the channel length (d) are plotted. Bottom-gate top-contact devices are realized as illustrated in the sketches on the left.

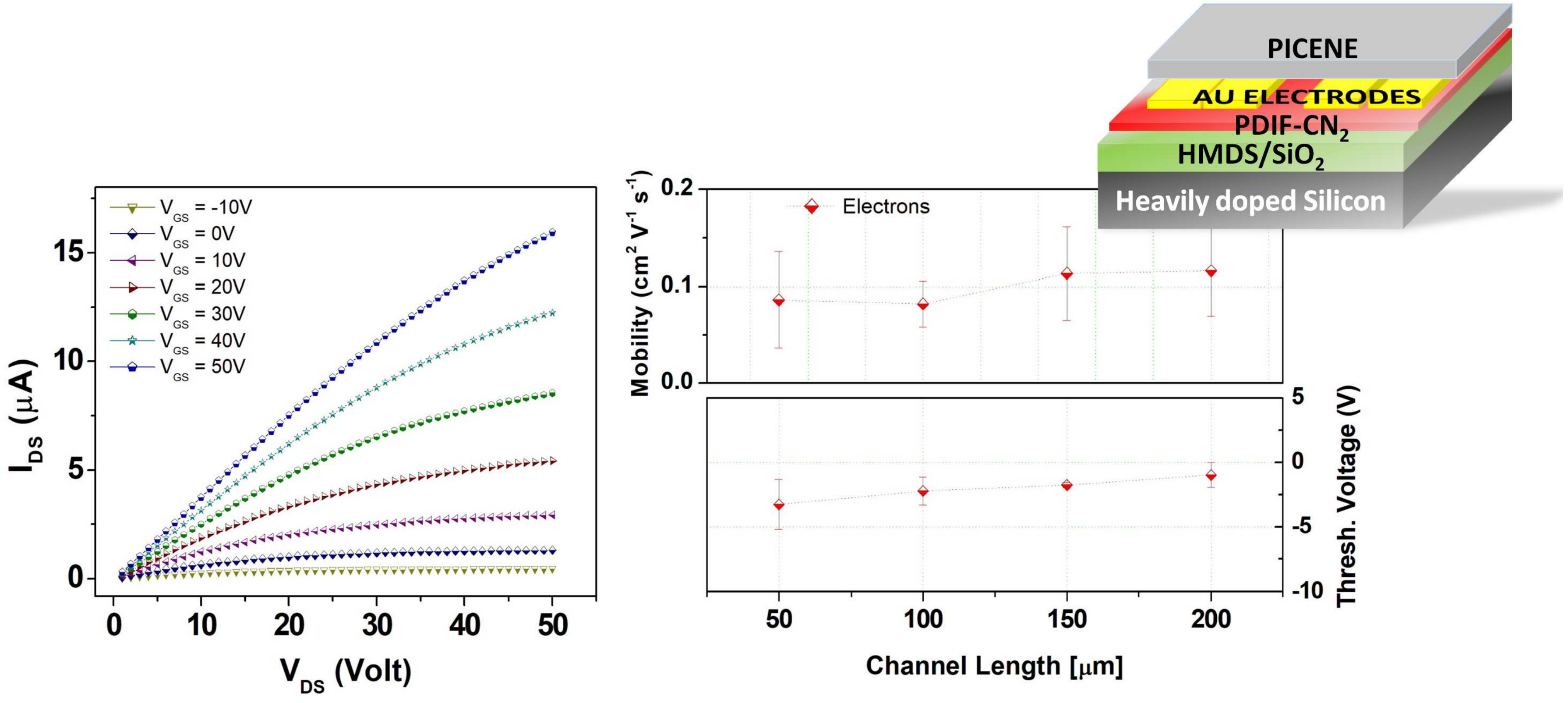

Figure S4 Middle-contact bottom-gate devices are realized as illustrated in the sketch. N-type output curves measured for the heterostructure-based FET with thicknesses of PDIF-CN_2 15 nm and picene 60 nm, are reported for different gate voltages. For sake of clarity, only the output curves, measured for a device with *L*=150 μm, are here reported. On the right panel, are plotted the main mobility and threshold voltage values of the picene/PDIF-CN_2 devices for the different channel lengths (*L*). No *p*-type (hole accumulation phenomenon) characteristics have been observed.

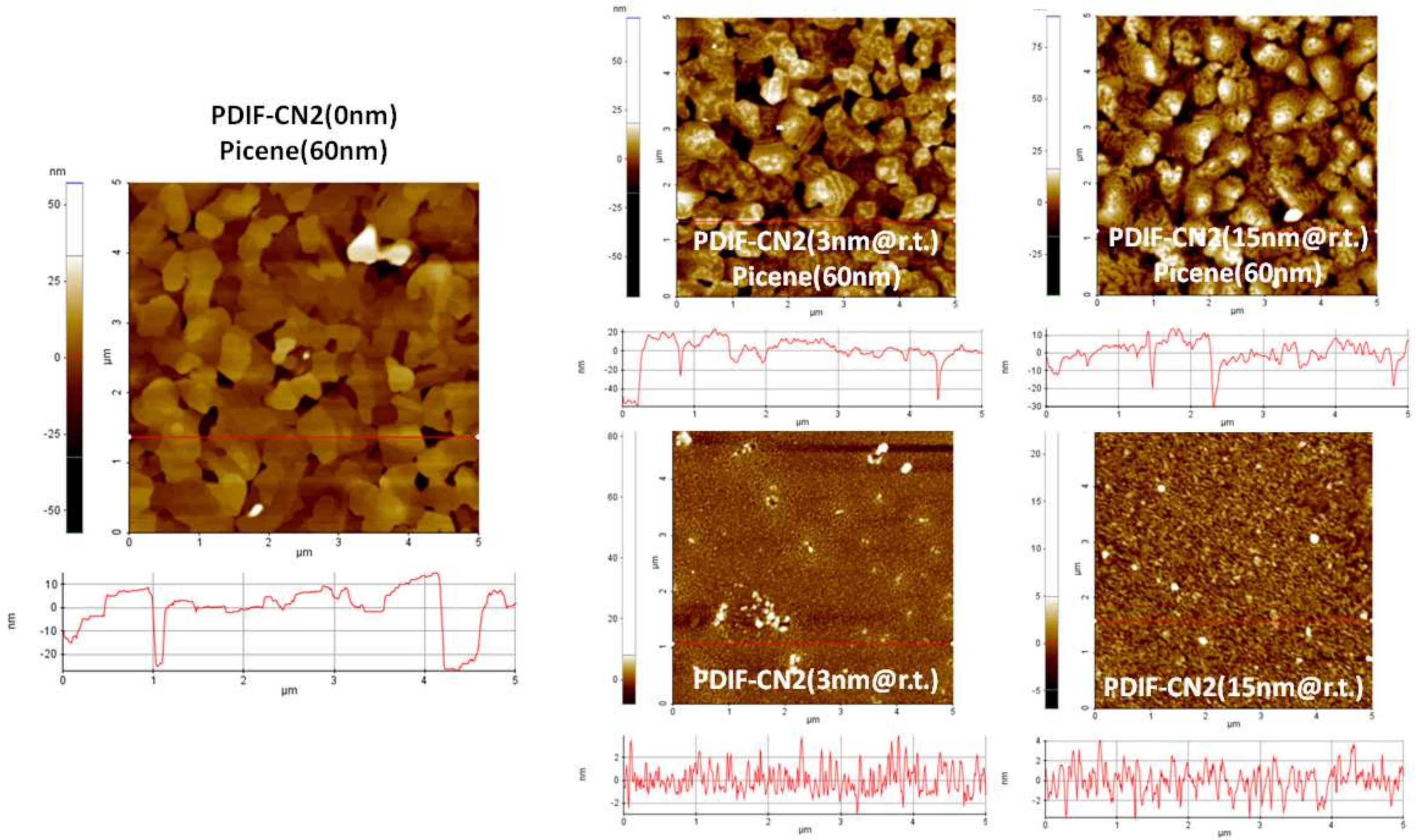

Figure S5 AFM 5x5 μm^2 images and topographic profiles of inverted PDIF-CN_2/picene heterostructures are shown and compared with the morphology of a solo-picene film with thickness of 60 nm and PDIF-CN_2 thin films (3 nm and 15 nm of thickness) deposited at room temperature directly on SiO_2/HMDS substrate.

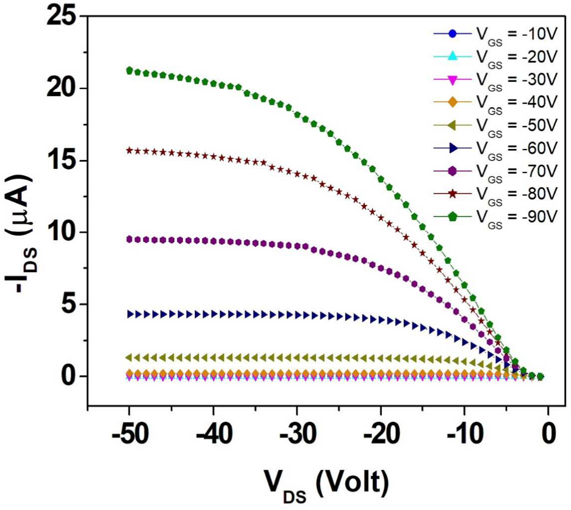

(a)

Picene(60nm)		
PDIF-CN2	Mob(P)$_{Ave.}$	Vth(P)$_{Ave.}$
0nm	0.791	-50.7
3nm	0.601	-31.6
15nm	0.522	-34.7

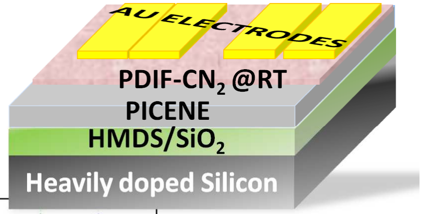

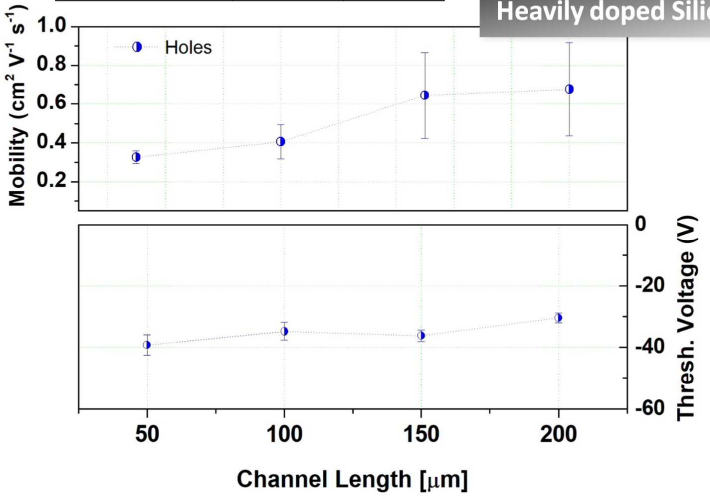

(b)

Figure S6 Inverted PDIF-CN$_2$/picene heterostructure was deposited to realize top-contact bottom-gate devices as illustrated in the sketch (both organic compounds were evaporated while the growth surfaces was kept at room temperature (22 °C)). (a) Output currents for *p*-type charge carriers transport for the heterostructure-based FET composed of a picene film of thickness of 60 nm covered with a PDIF-CN$_2$ film of thickness of 15 nm is plotted for different gate voltages. For sake of clarity, here we report only the output curves of a device with *L*=150 μm. The main mobility and threshold voltage values of the picene/PDIF-CN$_2$ devices as a function of *L* are plotted (b). In the table, are listed the full-average mobility and threshold voltage of solo-picene device, PDIF-CN$_2$/picene (3 nm / 60 nm) and PDIF-CN$_2$/picene (15 nm / 60 nm). No *n*-type characteristics (electron accumulation phenomenon) have been observed.

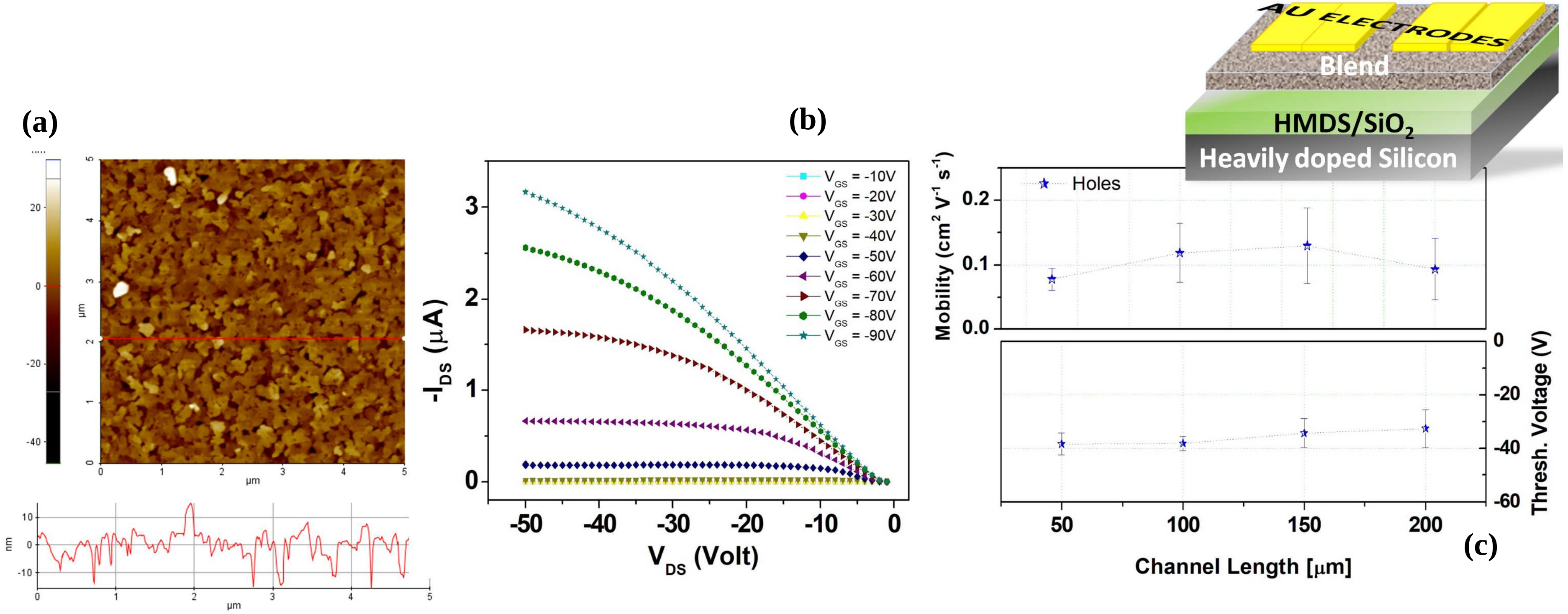

Figure S7 Characterization of picene/PDIF-CN_2 blend with a dilution ratio 1/6. Materials were deposited contemporarily keeping the substrate at room temperature (22°C) for an overall film thickens of 70 nm. Top-contact bottom-gate devices are realized as illustrated in the sketch. (a) AFM image of a 10 x 10 μm^2 scanning area and line scan, (b) output curves for *p*-type accumulation in a device of 150 μm channel length, (c) mean mobility and threshold voltages for different channel lengths are illustrated. No *n*-type response was observed. Mobility and threshold voltage result reduced if compared with a picene single-layer device (see Figure S1 for comparison).

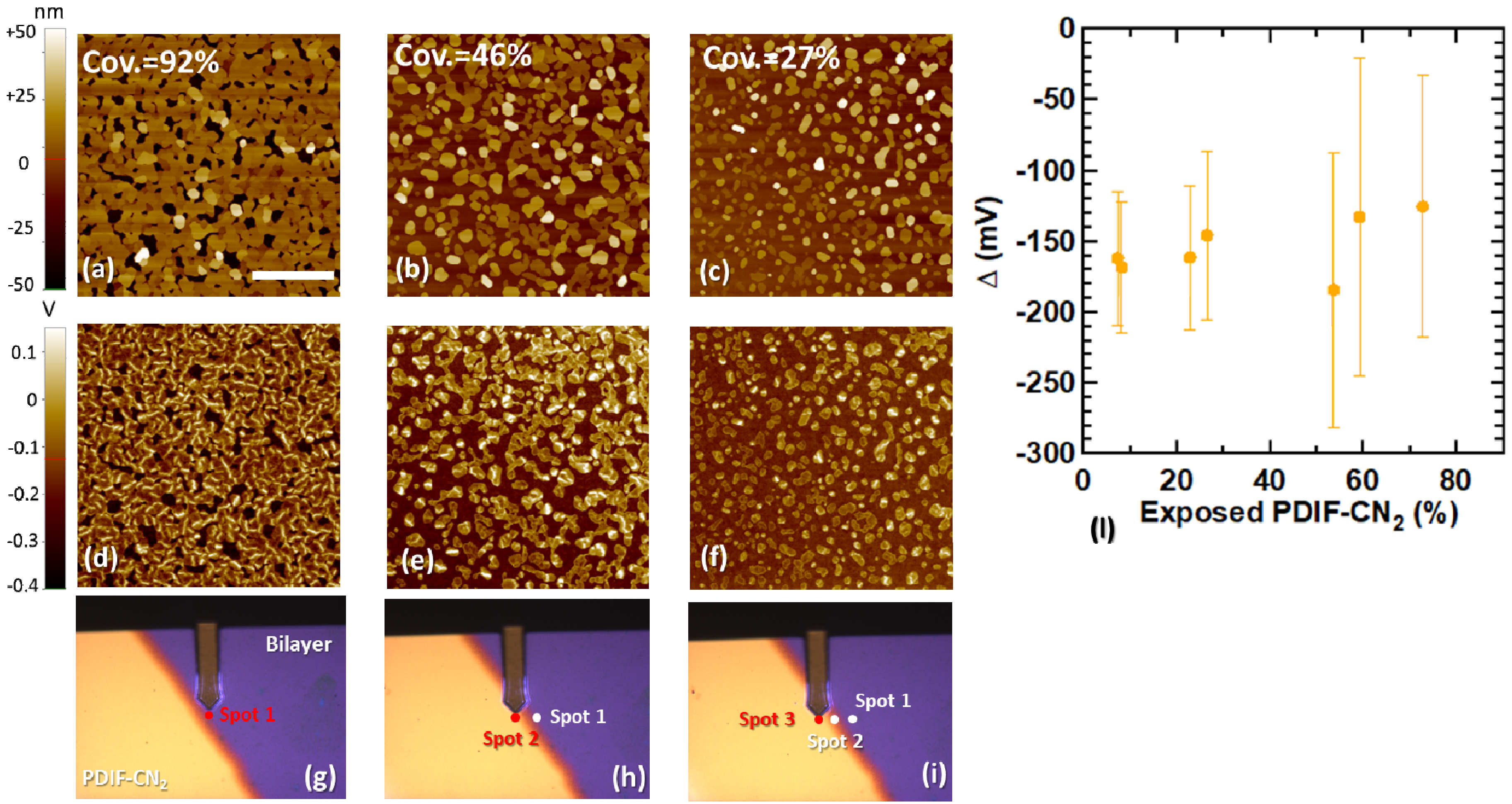

Figure S8 Picene/PDIF-CN_2 Δ values plotted as function of the exposed PDIF-CN_2 surface (l). Non-contact topographies of picene (20 nm)/PDIF-CN_2 (10 nm) for different picene coverage. (d)-(f) Corresponding surface potential obtained via SKPM (a)-(c).

Different picene coverage was obtained progressively reducing the nominal thickness of the picene film as illustrated considering areas progressively farther from the picene film boundary due to a shadow effect (note: during the deposition of the picene film, part of the PDIF-CN_2 underlayer was protected with a metallic mask) (g)-(i).

REFERENCES

[1] Chiarella, F., Perroni, C.A., Chianese, F. et al. Post-Deposition Wetting and Instabilities in Organic Thin Films by Supersonic Molecular Beam Deposition. Sci. Rep. **2018**, 8, 12015.
